# Vaginal Isolates of Candida glabrata Are Uniquely Susceptible to Ionophoric Killer Toxins Produced by Saccharomyces cerevisiae

**DOI:** 10.1128/AAC.02450-20

**Published:** 2021-06-17

**Authors:** Lance R. Fredericks, Mark D. Lee, Hannah R. Eckert, Shunji Li, Mason A. Shipley, Cooper R. Roslund, Dina A. Boikov, Emily A. Kizer, Jack D. Sobel, Paul A. Rowley

**Affiliations:** a Department of Biological Sciences, University of Idaho, Moscow, Idaho, USA; b Department of Internal Medicine, Division Infectious Diseases, Wayne State University School of Medicine, Detroit, Michigan, USA

**Keywords:** *Candida glabrata*, *Saccharomyces*, antifungals, azole, candidiasis, killer toxins, polyene, vulvovaginal

## Abstract

Compared to other species of *Candida* yeasts, the growth of Candida glabrata is inhibited by many different strains of *Saccharomyces* killer yeasts. The ionophoric K1 and K2 killer toxins are broadly inhibitory to all clinical isolates of C. glabrata from patients with recurrent vulvovaginal candidiasis, despite high levels of resistance to clinically relevant antifungal therapeutics.

## INTRODUCTION

Vulvovaginal candidiasis (VVC) is estimated to afflict two in every three women worldwide at some point in their lives, causing significant suffering and associated economic losses (1–3). Candida albicans is most often isolated as the dominant species present in cases of VVC, followed by Candida glabrata (4). In certain diabetic patient populations, C. glabrata can be the dominant yeast species associated with VVC (5, 6). The main treatment for VVC is the orally administered fungistatic azole fluconazole. During pregnancy, to relieve symptoms of VVC and prevent *Candida-*associated complications, topical application of azoles is preferred over oral administration due to the potential for fetal toxicity in the first trimester (7). However, drug resistance in isolates of C. glabrata and other species of *Candida* yeasts is increasing and can result in long courses of suppressive treatment and treatment failure (8–10). The limited availability of effective nontoxic therapies to treat VVC warrants the exploration of novel therapeutics that are active at the normal low pH of the vagina.

**FIG 1:**
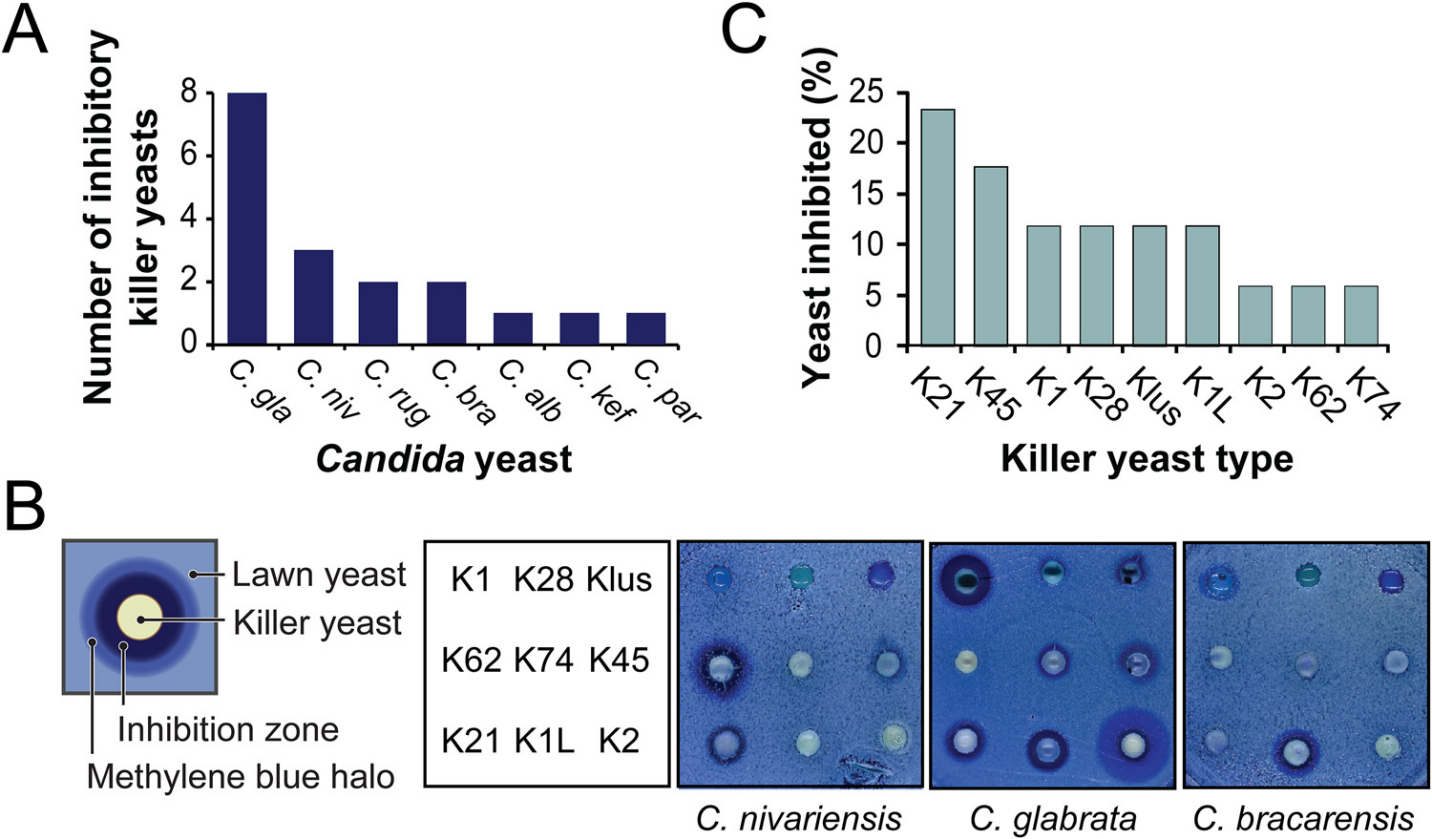
Candida glabrata is more susceptible to inhibition by killer yeasts than other species of *Candida* yeasts. (A) Number of killer yeasts that inhibit the growth of different species of *Candida* yeasts: C. glabrata (*C. gla*), C. nivariensis (*C. niv*), C. kefyr (*C. kef*), C. bracarensis (*C. bra*), C. rugosa (*C. rug*), C. pararugosa (*C. par*), and C. albicans (*C. alb*) (*n* = 2). (B) Schematic illustration of the effect of a killer yeast on the growth of a competing lawn of yeast. Representative well assay plates with nine different killer yeasts on agar seeded with representative species of *Candida* yeasts are shown. (C) Percentage of *Candida* yeast species found to be inhibited by each type of killer toxin (*n* = 2).

Killer toxins produced by *Saccharomyces* “killer” yeasts are optimally active under acidic conditions (pH ≤4.6) that overlap the pH of the vaginal mucosa (pH ∼4.2) (11). In addition, there have been many studies describing killer yeasts that can inhibit the growth of pathogenic fungi (12–17). Given the discovery of novel killer toxins produced by *Saccharomyces* killer yeasts, 16 species of the *Candida* genus were screened for their susceptibility to nine killer yeast strains known to express K1, K1L, K2, K21/K66, K28, K45, K62, K74, or Klus killer toxins encoded by double-stranded satellite RNAs (18–24). To test the ability of different killer yeasts to inhibit the growth of *Candida* yeasts, a well assay was used to inoculate killer yeasts into killer assay agar plates (yeast extract-peptone-dextrose [YPD] agar with 0.003% [wt/vol] methylene blue buffered to pH 4.6 with sodium citrate [25]) seeded with ∼1 × 10^5^ *Candida* yeast cells (see Fig. S1 in the supplemental material). After 3 days of incubation at room temperature, growth inhibition was identified by the appearance of yeast-free zones and/or halos of oxidized methylene blue around killer yeasts (Fig. 1 and Fig. S1). Of the 16 species of *Candida* yeasts challenged, C. glabrata appeared to be the most susceptible to killer yeasts and was resistant only to the killer yeast that expressed K62 (Fig. S1 and Table S1). One additional species of the *Nakaseomyces* clade (Candida nivariensis) was also more susceptible to *Saccharomyces* killer yeasts than other *Candida* yeasts (Fig. 1A and B). The K21 killer yeast appeared to have the broadest spectrum of antifungal activity (Fig. 1C).

**FIG 2:**
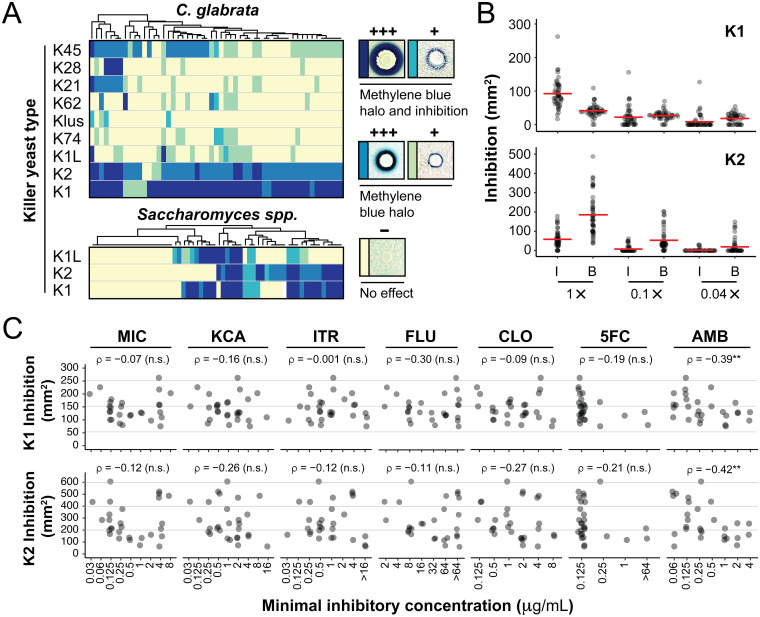
Drug-resistant clinical isolates of C. glabrata from the vagina are most susceptible to the ionophoric killer toxins K1 and K2. (A) Cluster analysis of the susceptibility of 53 isolates of C. glabrata (top) and 53 strains of *Saccharomyces* species (bottom) to different types of killer yeasts as assayed on agar plates. (B) Susceptibility of 50 isolates of C. glabrata to partially purified K1 and K2 killer toxins showing the mean killer toxin activity based on the area of complete growth inhibition (I) or methylene blue staining (B) on agar. The 1× concentration of K1 used was 13 μg/ml as measured by Western blotting using a custom antibody raised to a K1-derived peptide. (C) MICs of seven antifungal drugs against 27 Wayne State clinical isolates of C. glabrata compared to the total area (methylene blue staining and zone of growth inhibition) of cytotoxicity caused by K1 or K2 killer toxins. Correlations and significance values were calculated by Spearman’s rank correlation analysis (n.s., not significant; **, *P* < 0.05). Antifungal drugs assayed were fluconazole (FLU), clotrimazole (CLO), ketoconazole (KCA), miconazole (MIC), itraconazole (ITR), amphotericin B (AMB), and flucytosine (5FC).

As killer toxin susceptibility can vary widely within a species, 53 unselected clinical isolates of C. glabrata from the human vagina were challenged by killer yeasts. These clinical isolates were collected at the Wayne State University vulvovaginitis clinic in Detroit, MI, between 2015 and 2019. Killer yeasts were inoculated onto the surface of killer assay agar plates seeded with lawns of C. glabrata and qualitatively assayed for evidence of growth inhibition. Of the 477 interactions measured between killer yeasts and C. glabrata, K1, K2, and K45 killer yeasts inhibited the growth of 100%, 96%, and 75% of C. glabrata isolates, respectively (Fig. 2A, top). The remaining killer yeasts each inhibited <33% of C. glabrata isolates. The susceptibility of C. glabrata to K1 and K2 killer yeasts greatly contrasts the widespread resistance of *Saccharomyces* yeasts (Fig. 2A, bottom). To test the susceptibility of the clinical isolates of C. glabrata with acute killer toxin exposure, K1 and K2 toxins were partially purified from 1 ml of spent culture medium, as described previously (23). Exposure of lawns seeded with ∼1 × 10^5^ C. glabrata cells to K1 or K2 demonstrated concentration-dependent growth inhibition of C. glabrata (Fig. 2B). All isolates of C. glabrata were inhibited by K1 and K2 at the highest concentration tested, with K2 exposure resulting in large halos of methylene blue (185.68 ± 28.66 mm^2^ [95% confidence interval {CI}]), whereas K1 produced larger zones of growth inhibition (92.34 ± 11.24 mm^2^ [95% CI]) (Fig. 2B). The inhibition of C. glabrata is similar to the fungicidal effects of K1 and K2 toxins against susceptible strains of Saccharomyces cerevisiae (Fig. S2). Neither K1 nor K2 was acutely cytotoxic to cultured human cells at physiological pH as measured by an alamarBlue viability assay (Fig. S3). Both K1 and K2 retained measurable activity against yeast cells after a 1-h incubation with human epithelial cells (HeLa cell line) in Dulbecco’s modified Eagle medium with 10% serum at pH 7 (Fig. S3).

Clinical isolates of C. glabrata were found to vary in their resistance to antifungals used to treat both VVC and invasive candidemia using the NCCLS M27-A method (50) to calculate MIC values (Fig. 2C) and the disk diffusion assay (Fig. S2). Even when C. glabrata isolates were highly resistant to clinical antifungals, they remained susceptible to acute K1 and K2 exposure. There was no significant correlation between drug resistance and killer toxin susceptibility (Fig. 2C and Fig. S2), except for a weak correlation between K1 and K2 resistance and amphotericin B resistance (*P* < 0.05) (Fig. 2C and Fig. S2).

Killer toxins are notoriously strain and species specific to the point that they have been used to identify different strains of pathogenic yeasts (26). However, the data presented in this study show that most types of known *Saccharomyces* killer yeasts can inhibit the growth of C. glabrata. Specifically, the qualitative screening of killer yeasts identified that the ionophoric toxins K1 and K2 were broadly inhibitory to vaginal isolates of C. glabrata and that purified toxins inhibited growth in a concentration-dependent manner. The C. glabrata cell wall is structurally similar to that of S. cerevisiae, and this suggests that K1 and K2 bind the C. glabrata cell wall β-1,6-glucan as the primary receptor (27). C. glabrata also expresses a homolog of S. cerevisiae Kre1p, which is the glycosylphosphatidylinositol (GPI)-anchored secondary membrane receptor used by both K1 and K2 that enables membrane attack (28). The mechanism of C. glabrata intoxication is likely to involve the disruption of ion homeostasis by pore formation in the plasma membrane, as has been shown for S. cerevisiae (29). However, cell wall binding and the presence of Kre1p are not sufficient for intoxication, as K1 is able to bind the cell wall and utilize Kre1p of C. albicans, which is intrinsically K1 resistant (30, 31). Furthermore, it is unclear why other species of the *Nakaseomyces* genus (C. nivariensis and C. bracarensis) that are closely related to C. glabrata are more resistant to inhibition by the same killer yeasts.

The alteration of ergosterol biosynthesis can cause resistance to azoles and amphotericin B in *Candida* yeasts (32–35), and we find that the latter is significantly correlated with increased K1 and K2 resistance (Fig. 2C). As amphotericin resistance can be caused by a reduction in the concentration of membrane ergosterol, increased K1 and K2 resistance in C. glabrata could be due to alterations in the composition, fluidity, and permeability of the yeast plasma membrane. Similar protection from K1 intoxication is observed in strains of S. cerevisiae with defects in ergosterol biosynthesis (36). Natural K1-resistant strains of S. cerevisiae also have lower expression levels of ergosterol biosynthesis genes and reduced concentrations of ergosterol esters (37). The depletion of membrane sterols can result in resistance to many cytotoxic proteins (38–42). Moreover, sterol-rich membrane microdomains (lipid rafts) provide a nucleation point for protein toxins that bind raft-localized receptors (43–45). The depletion or redistribution of cholesterol can disrupt raft integrity and inhibit the binding of toxins to their cognate receptor (43, 46). Thus, the susceptibility of C. glabrata to K1 and K2 could be influenced by membrane ergosterol and the localization and function of the GPI-anchored secondary membrane receptor Kre1p.

The screening of killer yeasts has served to identify the unique activity of K1 and K2 against C. glabrata, suggesting that they could be useful as novel antifungal agents. Compared to azoles, these killer toxins are fungicidal, optimally active at low pH, and nontoxic to human cells at physiological pH (28, 47–49). Therefore, we speculate that with further mechanistic studies, formulation, and stabilization, K1 and K2 could be developed for topical application to combat C. glabrata associated with VVC.
